# Facile formulation and fabrication of the cathode using a self-lithiated carbon for all-solid-state batteries

**DOI:** 10.1038/s41598-020-68865-8

**Published:** 2020-07-16

**Authors:** N. Delaporte, A. Darwiche, M. Léonard, G. Lajoie, H. Demers, D. Clément, R. Veillette, L. Rodrigue, M. L. Trudeau, C. Kim, K. Zaghib

**Affiliations:** Center of Excellence in Transportation Electrification and Energy Storage, Hydro-Québec, Varennes, QC J3X 1S1 Canada

**Keywords:** Electrochemistry, Energy

## Abstract

We propose a innovative concept to boost the electrochemical performance of cathode composite electrodes using surface-modified carbons with hydrophilic moieties to increase their dispersion in a Lithium Nickel Manganese Cobalt Oxide (NMC) cathode and in-situ generate Li-rich carbon surfaces. Using a rapid aqueous process, the hydrophilic carbon is effectively dispersed in NMC particles followed by the conversion of its acid surface groups (e.g. –COOH), which interact with the NMC particles due to their basicity, into grafted Li salt (–COO^−^Li^+^). The solid-state batteries prepared using the cathode composites with surface-modified carbon exhibit better electrochemical performance. Such modified carbons led to a better electronic conduction path as well as facilitating Li^+^ ions transfer at the carbon/NMC interface due to the presence of lithiated carboxylate groups on their surface.

## Introduction

The increase in energy demand and environmental concerns has accelerated the development of sustainable energy storage systems^1^. Lithium-ion batteries (LIBs) dominate the rechargeable battery market in the portable electronic devices and electric vehicles sector because of their high energy density and long-life^2^. However, all-solid-state batteries (ASSB) are being reconsidered due to their higher energy density and the absence of inflammable liquid electrolyte, which makes them safer^3^. Further, the need for energy storage devices with higher energy density requires high capacity cathode materials, such as Ni-rich Lithium Nickel Manganese Cobalt Oxide (NMC) (LiNi_x_Mn_1−x−y_Co_y_O_2_, x ≥ 0.8, y ≤ 0.1)^4^. The Ni-rich NMC material contains high surface concentrations of LiOH and Li_2_CO_3_ originating from the residual Li precursors and reactions with humid air^5^. As these species are detrimental for the stability of the polymer electrolyte in a battery, it is necessary to remove them; this may be achieved by additional washing^6^. The problem is that the additional washing and drying steps add up significant process cost. Moreover, in ASSB, the ionic and electronic conduction occurs through solid phases, which is accomplished by the continuous percolation of particles across the electrode. Thus, achieving good dispersion of carbon and active material is important in ASSB as well as the removal of basic species at the surface of the NMC material.

Electrode fabrication can be achieved using different techniques such as three-dimensional printing^7^, spin coating^8^, paste generating method^9^, freeze-casting^10^, electrospinning^11^, solvent-based electrostatic spray deposition^12^, solvent-free dry powder coating^13^, pulsed laser deposition^14^, sputtering deposition^15^, dry painting^16^, screen printing^17^, filtration^18^, extrusion^19^ or recently the solvent-free roll-to-roll manufacturing technique^20^. However, the most commonly used fabrication technique is the so-called web-coating method or doctor-blading that consists to spread an electrode slurry on a current collector foil^21^. The viscosity of the ink, the nature of carbons^22^, binders and active materials, as well as the solvents used for the dispersion strongly impacts the porosity and the roughness of the electrode.

Several efforts have been devoted to improve the quality of the electrodes leading to better electrochemical performance. For instance, dispersants are widely employed to help dispersing the carbon and the active material in electrode films. Kil et al*.* reported the utilization of polyurethane-based dispersants to increase the dispersion of carbon black slurries^23^. Similarly, Zhi-an et al*.* proposed to disperse Super P carbon in polyacrylic acid dispersant with a high-shear mixer and to combine this suspension with a standard binder and LiFePO_4_ (LFP) cathode material to make an electrode^24^. Polyacrylate surfactant orotan has been also reported for dispersing carbon black into LiCoO_2_ cathode^25^. Triton X100 dispersant is one of the most famous and most employed to help making electrodes, it was recently employed for the dispersion of argyrodite solid-state electrolyte in the composition of NMC cathodes^26^. This non-ionic dispersant was also used for the preparation of aqueous slurries to make LFP electrodes for instance^27^. Polyethyleneimine^28^, sodium dodecylesulphate (SDS) or exadecyltrimethylammonium bromide (CTAB)^29^ dispersants were also employed to favor the dispersion of LFP cathode material in aqueous slurries. Dominko et al*.* reported an innovative method to uniformly disperse carbon black into cathode films^30^. The active material is firstly treated with a gelatin solution and then, the adsorbed gelatin controls the deposition of carbon black that led to its uniform distribution in the final composite. Another strategy to enhance the contact between the active material of an electrode and the carbon additive is to load the active material in a carbon template such as CMK-3 and CMK-8 mesoporous carbons^31^, vertical aligned carbon nanotubes (CNT)^32^ or again CNT particles host containing spherical macropores^33^. Mechanical techniques have been also employed to enhance the dispersion of active materials and carbons. Highly dispersed Ketjen black carbons were obtained using ball milling technique in solvents, modifying the particle sizes and structures of the carbons and resulting in improved dispersions^34^. The high intensive shear mixing device Nobilta was often employed to produce intimate contact between the active material and the carbon through a dry mixing process^35^.

In this study, we introduce the new concept of “Li-rich carbon”. A facile modification of carbon conductive agents is proposed to enhance the dispersion of carbon inside the cathode electrode and neutralize the basicity of Ni-rich NMC cathode material. This approach reduces the electrochemical resistance of the electrode by developing better electronic conduction path as well as facilitating Li^+^ ions transfer at the carbon/NMC interface due to the presence of lithiated carboxylate groups on the surface of carbon. In addition, the chemical modification of the high surface area carbon (e.g. Ketjen black carbon) strongly reduces the micro- and meso-porosity, avoiding a too high uptake of solvent during the electrode preparation, thus facilitating its fabrication and yielding homogeneous and smooth electrodes after drying step. Finally, such modified carbons are better dispersed in the PEO-based polymer in comparison to the pristine carbons leading to a better quality of electrode.

## Results

### Preparation of the in-situ generated Li-rich carbons

**Figure 1:**
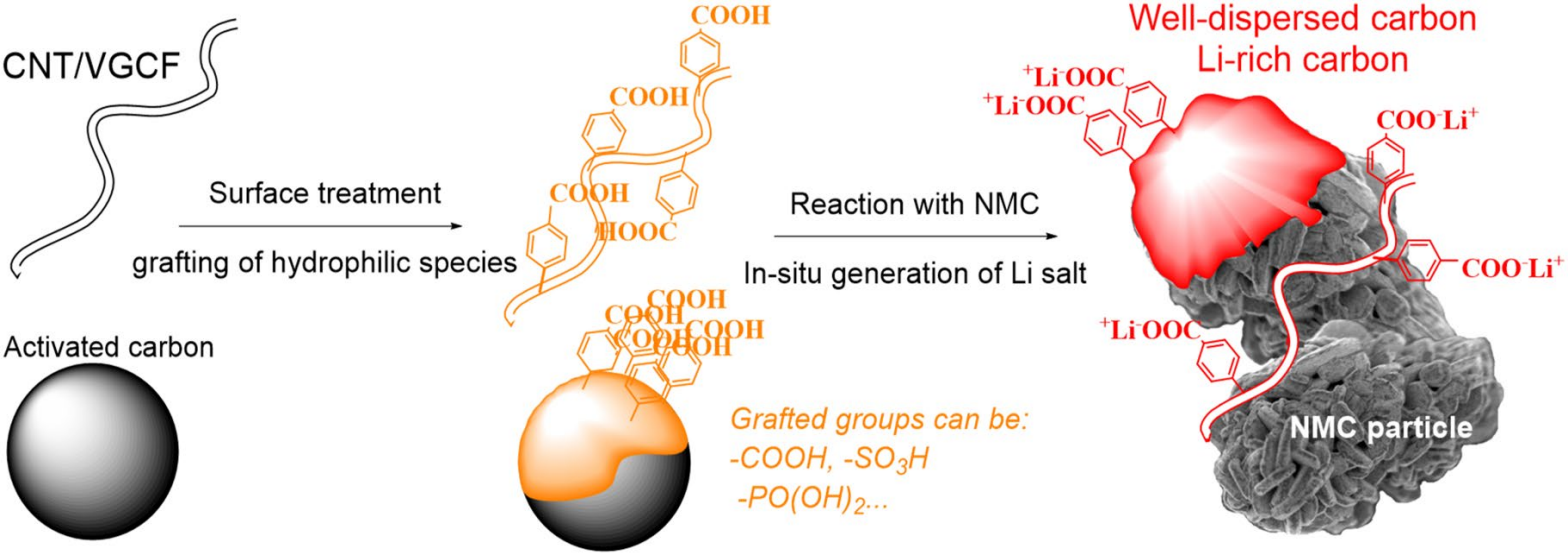
Schematic illustration of the reaction procedures: the first step (in orange) is to graft hydrophilic molecules (different groups can be used) on carbon surface (e.g. CNT, Ketjen Black) and the second step (in red) is to in-situ generate Li salts on carbon in the presence of NMC particles.

**Figure 2:**
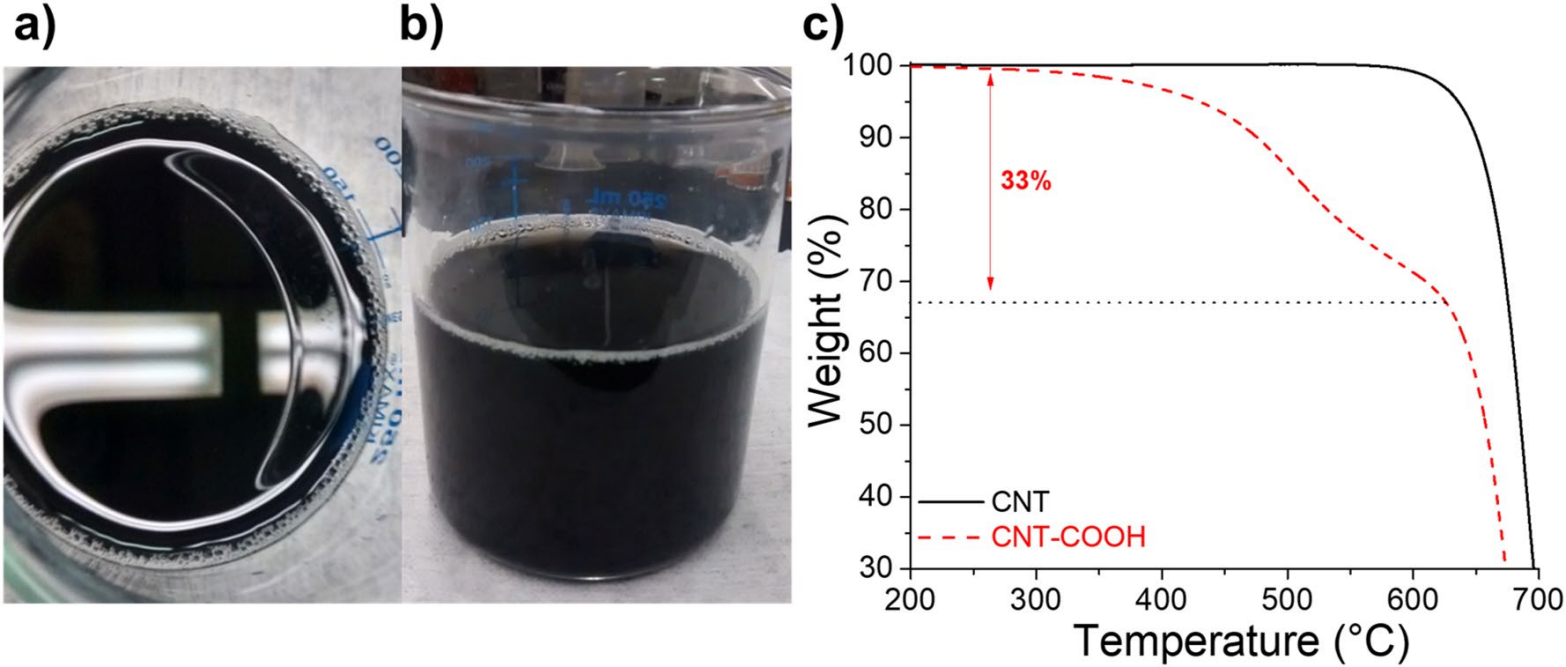
(**a**,**b**) Photographs of a suspension of CNT–COOH in deionized water, and (**c**) Thermogravimetric curves for pristine CNT (—) and CNT-COOH (red dashed line) carbons.

To address these problems, we propose a new concept using surface-modified carbons with hydrophilic moieties, as illustrated in Fig. 1. The modification procedure of different carbons (vapor grown carbon fiber, VGCF; carbon nanotubes, CNT; Ketjen Black) is described in the “Methods” section, which is adapted from recent research works of our group^36,37^. The modified carbon is first dispersed in deionized water to obtain a perfect and homogeneous suspension, as shown in Fig. 2a,b, for CNT–COOH^38^. Further, an excess NMC (99 or 99.5 wt% of the total solid mass) is added to the solution and mixed thoroughly. Here, the pH of the solution rapidly increases due to the LiOH and Li_2_CO_3_ present on the surface of NMC particles, which induce the immediate conversion of acid surface groups (aryl–COOH) to grafted Li salt (aryl–COO^−^Li^+^) on carbon (Fig. 1), leading to the “Li-rich carbon” deposited on the surface of NMC particles.

### Characterization of NMC@carbon electrode composites

Figure 2 shows the photographs of an aqueous suspension of CNT–COOH after dispersing with an ultrasonic tip. Generally, due to the sp^2^-like carbon atoms constituting the graphitized structure of nanotubes, the pristine CNT before treatment is barely dispersed in water. In addition, strong agglomeration is often observed because of interlacement between each carbon nanotubes and π-stacking effect^39^. It is observed that after functionalization, a very stable suspension of CNT–COOH in water is present without visible agglomerates on the bottom of the beaker and residual carbon on the surface of water. This confirms the efficient grafting reaction with hydrophilic aryl–COOH groups (see Fig. 1). Similar results were obtained with VGCF–COOH and EG carbons. This first step is mandatory to achieve an optimized coverage of the NMC cathode material with the smallest quantity of carbon, which is often impossible with pristine carbon. Figure 2c shows the thermogravimetric curves for CNT (—) and CNT–COOH (red dashed line) carbons. While pristine CNTs are stable up to ~ 620 °C, the CNT–COOH carbon demonstrates a mass loss of ~ 33% between 300–620 °C attributed to the degradation of the grafted aryl–COOH groups. The progressive loss is often observed for materials modified by diazonium chemistry^40^ and due to the formation of multilayers^41^ that degrade progressively with increase in temperature.

**Figure 3:**
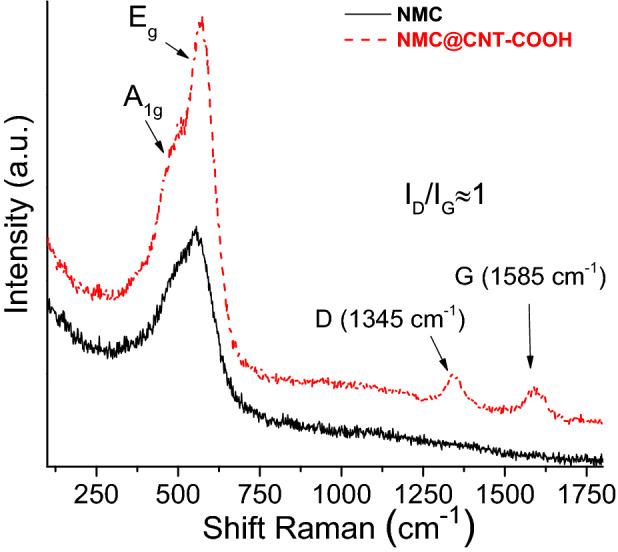
Micro-Raman spectra for NMC (—) and NMC@CNT–COOH (red dashed line).

Figure 3 presents the Raman spectra for the pristine NMC (—) and NMC@CNT–COOH composite material (red dashed line). Raman-active modes, A_1g_ and E_g_, of NMC crystal are observed for both samples around 500 and 560 cm^−1^, which correspond to the out-of-plane M–O stretch and in-plane O–M–O bend, respectively^42^. Additionally, small peaks at ~ 1,345 and ~ 1,585 cm^−1^ were observed for NMC@CNT–COOH powder that are attributed to the D (for disorder) and G (for graphitic) bands of the CNT–COOH carbon^43^. The D band is associated with disordered carbons or defective graphitic structures and its corresponding intensity increases with the amount of defaults in the carbon structure. The G band is assigned to the E_2g_ phonon of sp^2^-bonded carbon atoms, which is a characteristic feature of graphitic layers. The intensity ratio of the D to G bands (I_D_/I_G_) was high and nearly 1, while unmodified carbon nanotubes are normally characterized by a low I_D_/I_G_ due to the high graphitization level of these types of carbons^44^. This result reveals the presence of many defects on the surface of CNTs, which is consistent with the grafting of aryl–COOH moieties that involves the conversion of sp^2^ carbons to sp^3^ carbons via the creation of new C–C bonds^45^. This is generally reported for carbons modified with organic molecules via diazonium chemistry^46^. This observation is in accordance with the grafting yield of 33% estimated by thermogravimetric analyses (see Fig. 2c).

**Figure 4:**
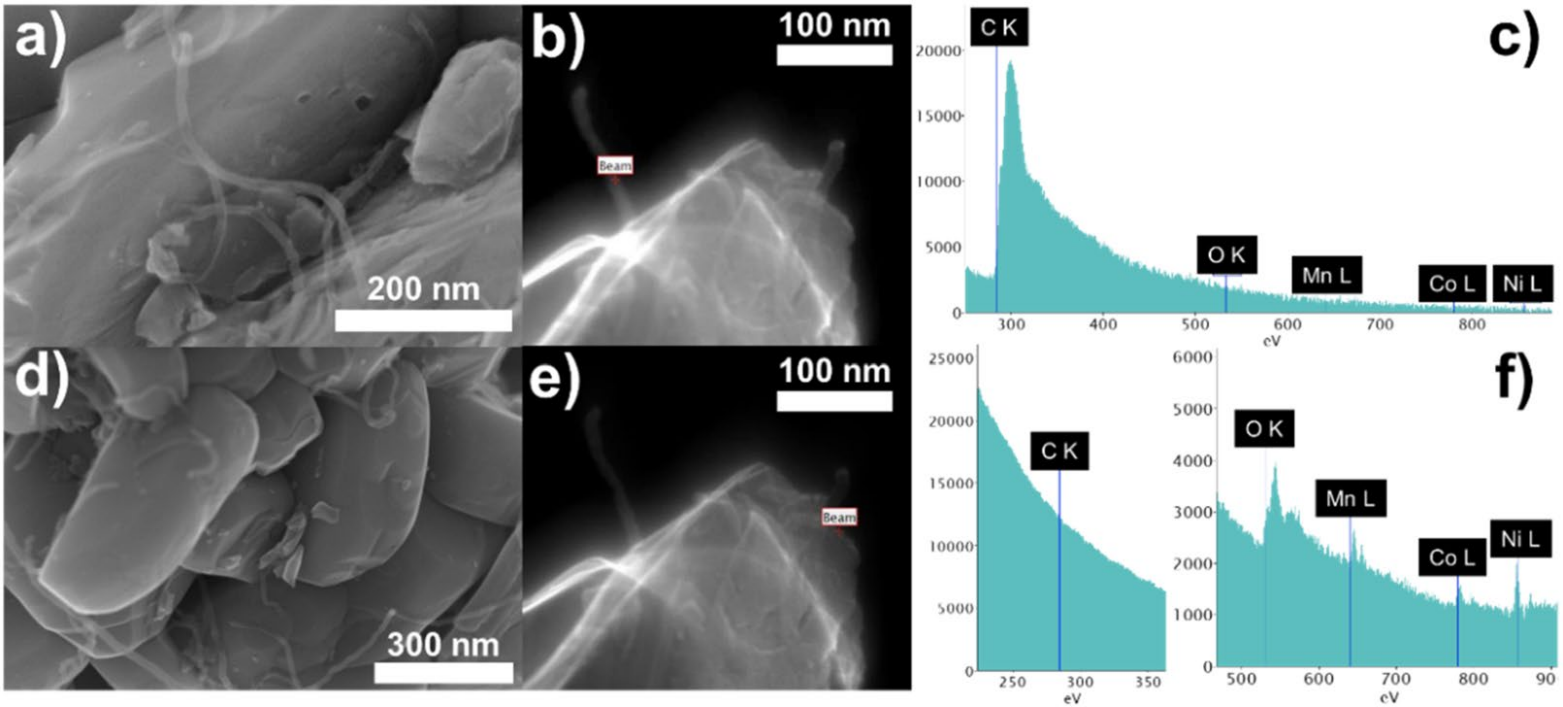
(**a**,**d**) SEM and (**b**,**e**) STEM-EELS images of NMC@CNT–COOH powder (representative spectrum images), and (**c**,**f**) corresponding STEM-EELS spectra.

Figure 4 shows the SEM and STEM-EELS images of the NMC@CNT–COOH powder. Modified carbon nanotubes sufficiently dispersed and attached on the surface of the NMC particles ensuring a good electrical contact. In contrary, the composite made by mixing the pristine NMC with the unmodified CNT showed large agglomerates of carbon fibers between NMC particles and poor electrical contact was obtained (see Supplementary Fig. S1 in the Supporting Information).

**Figure 5:**
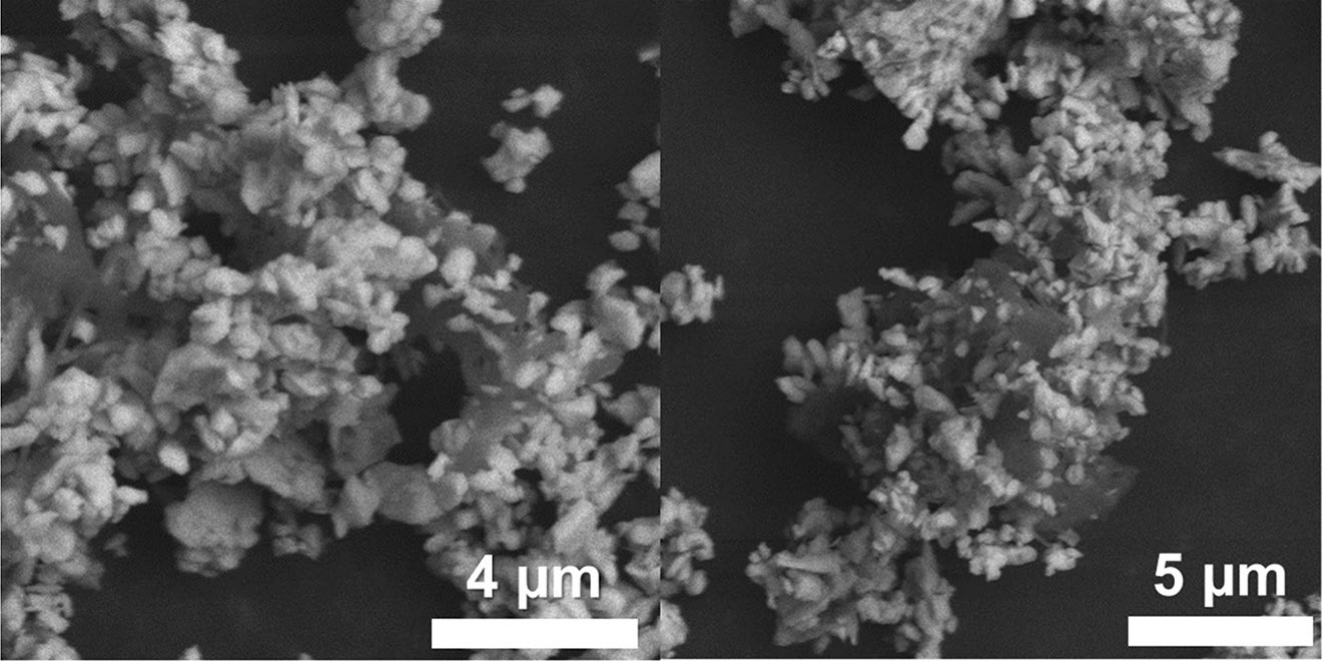
SEM images of NMC@VGCF–COOH powder.

Figure 5 shows SEM images of NMC@VGCF–COOH powder. Although VGCF carbon fibers are generally straight and measure several micrometers long, after grafting reaction and mixing with the NMC particles, a homogeneous mixture was observed with enhanced electron pathway in the composite electrode. In addition, the spontaneous conversion of aryl–COOH groups in Li-rich aryl–COO^−^Li^+^ moieties is considered to improve the Li^+^ ions transfer between the active material and polymer electrolyte. Recently, a polymer layer deposited on the surface of a lithium anode and composed of the same lithiophilic –COO^−^Li^+^ groups has been reported to favor a uniform Li-ion flux at the electrolyte/electrode interface, thus avoiding the dendrite formation^47^. Moreover, a PTCLi_4_-coated LTO material, with its surface extremely rich in –COO^−^Li^+^ moieties, was able to cycle at − 20 °C due to the increase of the Li-ion transfer at the electrode–electrolyte interface^48^. Finally, the carbon seems to be merged with the active material creating an intimate contact necessary for achieving good electrochemical performance. Similar result was noted with the cathode composite made with EG carbon.

**Figure 6:**
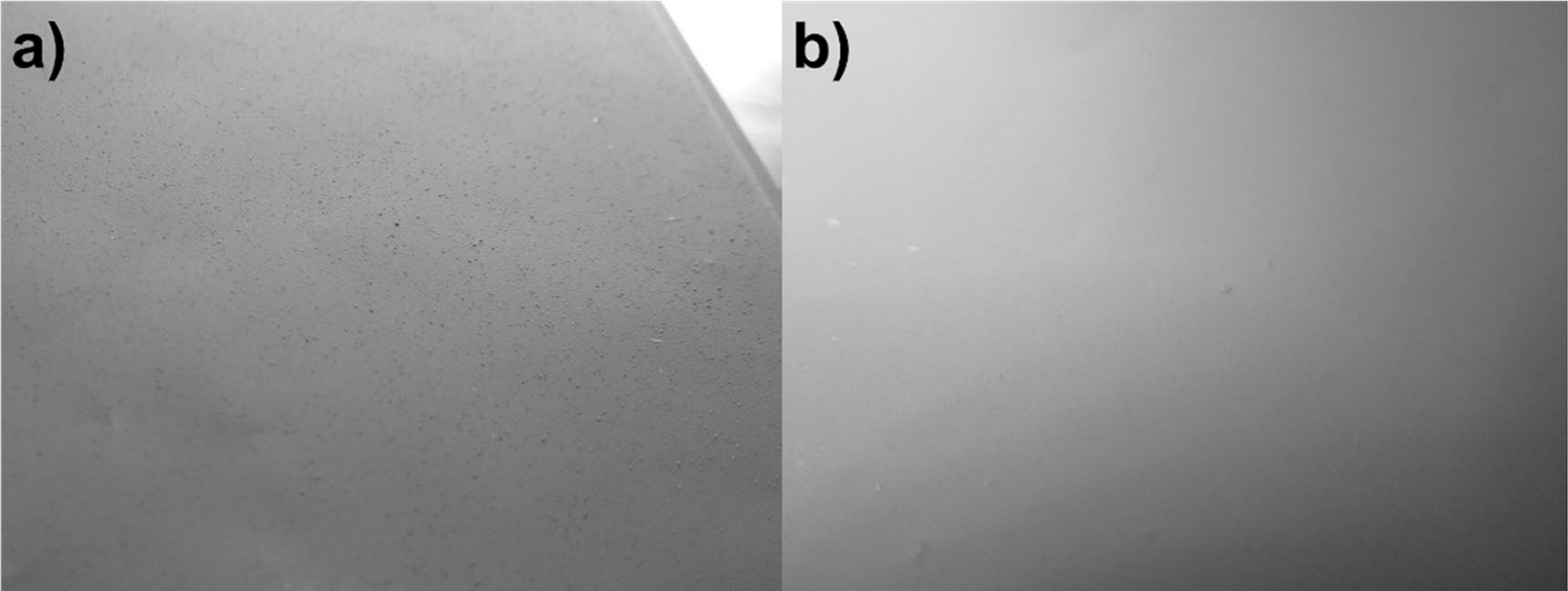
Photographs of NMC electrodes synthesized with (**a**) a mixture of unmodified NMC and Ketjen, and (**b**) a mixture of unmodified Ketjen and NMC@VGCF–COOH composite.

Finally, owing to the better distribution of carbon with the active material and affinity of such grafted groups with the polymer electrolyte, the quality of the electrode is considerably improved, as shown in Fig. 6. The electrode synthesized with a mixture of pristine NMC and unmodified Ketjen carbon (Fig. 6a) exhibited visible small agglomerates leading to a rough surface that causes bad surface contact with the solid polymer electrolyte. This is confirmed with the poor electrochemical performance recorded for this electrode, especially at high C-rates, as shown in Fig. 9 in the manuscript. In contrary, the electrode synthesized with the NMC@VGCF–COOH composite (Fig. 6b) presents a smoother surface with no or a negligible number of agglomerates. Consequently, superior electrochemical performances are obtained for this electrode, shown in Supplementary Fig. S5b (blue filled triangle).

**Figure 7:**
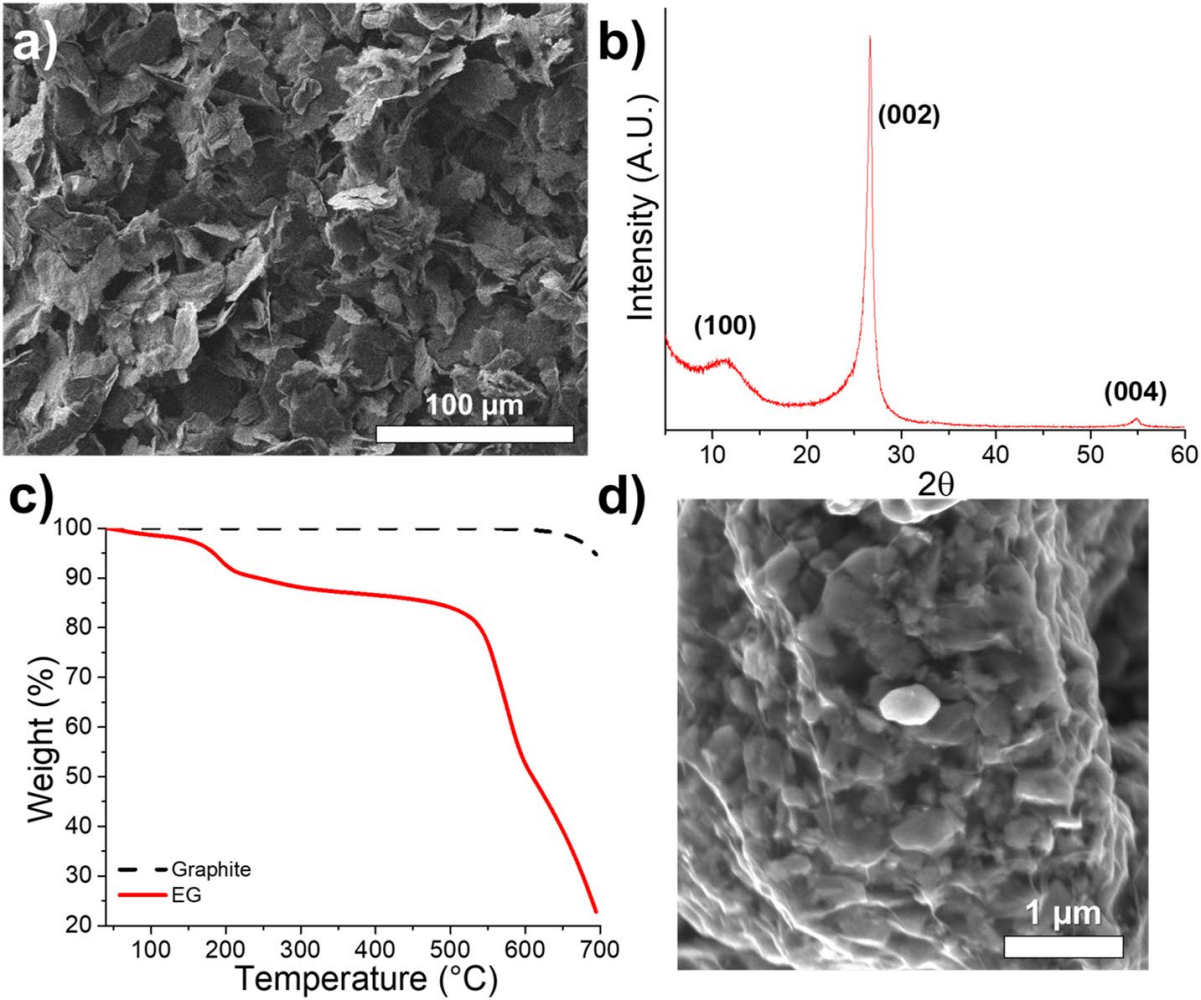
(**a**) SEM image and (**b**) XRD pattern of EG powder, (**c**) thermogravimetric curves of graphite and EG powders, (**d**) SEM image of NMC@EG powder.

Recently, we reported the use of exfoliated graphite (EG) to fabricate self-standing cathodes^18^. This carbon is composed of a few graphene sheets (Fig. 7a) and presents a high number (~ 12 wt%, see Fig. 7c) of oxygen molecules (e.g. –COOH) owing to a broad peak at approximately 2θ = 12°. The peak is caused by a graphite oxide material generated during the electrochemical exfoliation of the graphite foil. As shown in Fig. 7d, the graphene sheets are attached to the cathode material, enhancing electrical contact; further, the NMC particles are visible through the sheets. An additional SEM image is available in the Supporting Information (see Supplementary Fig. S2) showing a NMC particle of approximately 4 µm of diameter with a graphene sheet covering its entire surface.

**Figure 8:**
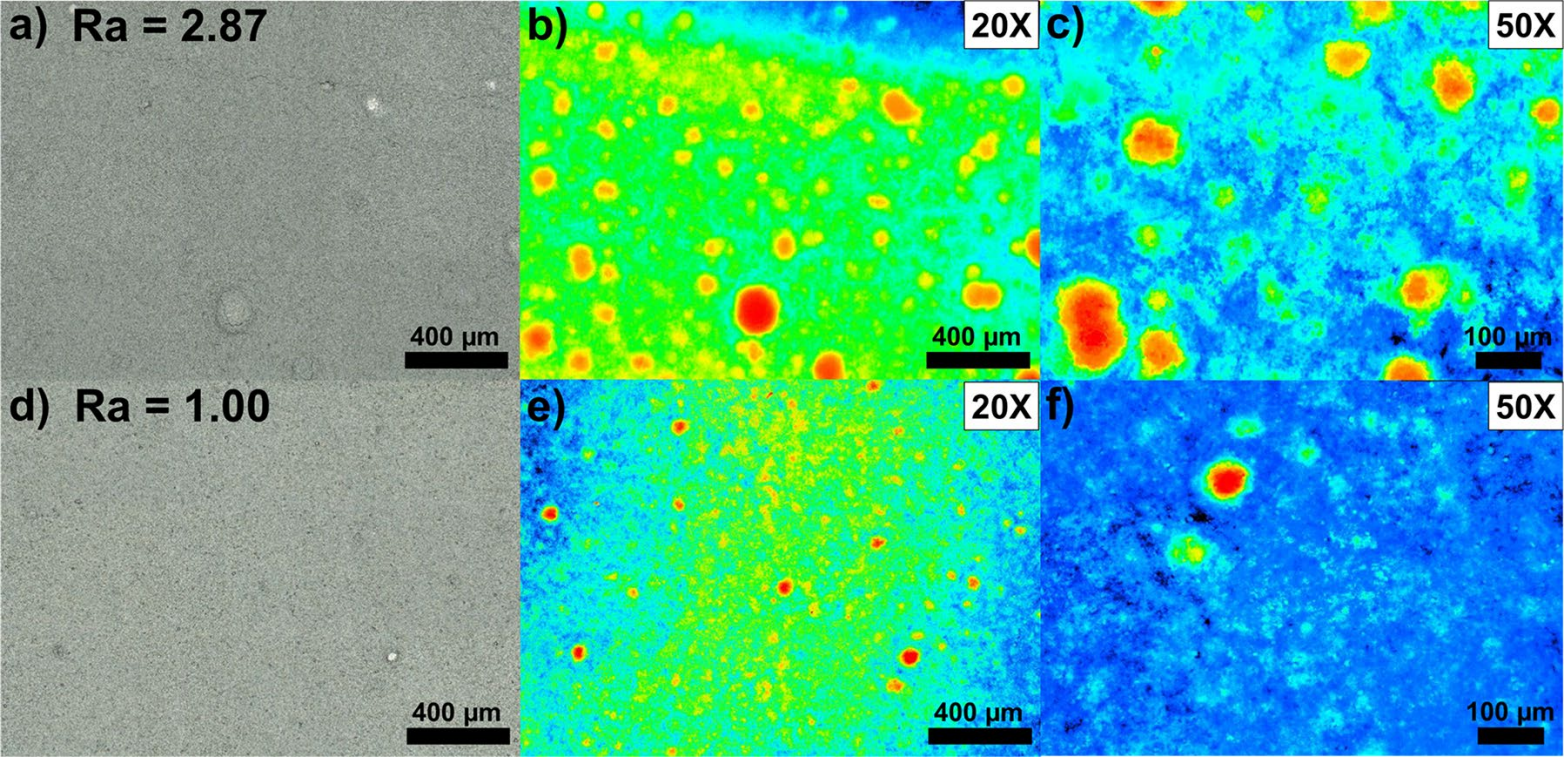
(**a**,d**)** Optical and (**b**,**c**,**e**,**f**) 3D confocal microscopy images of NMC electrodes made with: (**a**–**c**) pristine NMC and unmodified Ketjen Black carbon and (**d**–**f**) the NMC@VGCF–COOH composite with the modified grafted-Ketjen-acid carbon. Magnifications of × 20 (**a**,**b**,**d**,**e**) and × 50 (**c**,**f**) were used. The arithmetic mean roughness (Ra) is given for each electrode.

Additional tests were conducted with Ketjen Black carbon modified with NTCDA–(aryl)_2_ groups (Sections S2 and S3 in the Supporting information show the characterization and the electrochemical tests results). Here, better affinity of grafted moieties with the polyethylene oxide (PEO)-based polymer and the reduction of the micro- and meso-porosity yield homogeneous cathodes with less porosity induced during the drying process. In fact, carbons are known to adsorb a high quantity of solvent during the electrode preparation. After modification, a drop of the BET specific surface area from 1,238 to 600 m^2^ g^−1^ was observed due to the obstruction of small pores with the organic moieties. Thus, less solvent is adsorbed during mixing, which facilitates the electrode preparation and avoids the generation of porosity in the dry electrode caused by the continuous evaporation of solvent during drying step. Figure 8 shows the optical and corresponding 3D confocal microscopy images of NMC electrodes made with the pristine NMC and Ketjen black carbon (Fig. 8a–c) and with the NMC@VGCF–COOH composite and the modified grafted-Ketjen-acid carbon (Fig. 8d–f). It is worth noting that for the electrode made with the unmodified Ketjen black carbon, large agglomerates were observed everywhere on the surface of the cathode. They were identified by orange/red circles on the 3D confocal microscopy image with a × 20 magnification (see Fig. 8b). In contrary, using the NMC@VGCF–COOH composite combined with the grafted-Ketjen-acid carbon led to a smoother electrode, as put in evidence with the 3D confocal microscopy image with the same magnification (see Fig. 8e). In fact, less orange/red spots were observed on the map and their average size strongly diminished. The Ra value represents the arithmetic average of the roughness profile and more the value is low and more the surface is smooth without defaults. Ra values of 2.87 and 1.00 were obtained for the electrodes made by mixing NMC with Ketjen Black carbon and for the cathode prepared using the NMC@VGCF–COOH composite and the grafted-Ketjen-acid carbon, respectively. Thus, the electrode made with the modified carbons has the lowest roughness, which means it has a better uniformity with a flat surface. This result is consistent with the photographs of electrodes presented in Fig. 6.

### Electrochemical performance of NMC@carbon electrode composites

**Figure 9:**
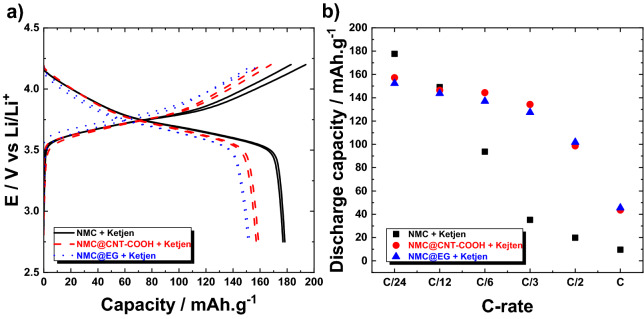
(**a**) Galvanostatic charge/discharge profiles at a cycling rate of C/24 and (**b**) rate capability of three NMC electrodes synthesized with NMC and Ketjen Black (—, filled square); NMC@CNT-COOH composite and Ketjen Black (red dashed line, red filled circle); and NMC@EG composite and Ketjen Black (blue dotted line, blue filled triangle).

Figure 9a shows the voltage profiles obtained at C/24 for the blank electrode synthesized with unmodified NMC and Ketjen Black (—) and the two cathodes fabricated with NMC@CNT–COOH (red dashed line) and NMC@EG (blue dotted line) composites. While the electrodes synthesized with NMC@carbon composites delivered a specific capacity of approximately 155–160 mAh g^−1^, the blank electrode presented a capacity of approximately 180 mAh g^−1^, which is higher than the anticipated capacity of NMC622 at 4.2 V cut-off (i.e. 160 mAh g^−1^ in polymer electrolyte). This higher capacity is primarily due to an underestimation of the active material mass caused by the inhomogeneity of the electrode and bad dispersion of the pristine Ketjen Black carbon and active material in the polymer matrix. Figure 9b presents the rate capability of the same electrodes. A similar discharge capacity of ~ 150 mAh g^−1^ was obtained for all the electrodes from low C-rates up to C/12. When the cycling rate increased, a rapid capacity fading was observed for the blank electrode (filled square), which delivered a poor discharge capacity of 35 mAh g^−1^ at C/3, while NMC@EG (blue filled triangle) and NMC@CNT-COOH (red filled circle) cathodes delivered a discharge capacity of 130 and 135 mAh g^−1^, respectively, at the same rate. This is because of the intimate contact between modified-carbons and NMC particles providing enhanced electron pathway in the composite electrode. Moreover, the spontaneous conversion of aryl–COOH groups in Li-rich aryl–COO^−^Li^+^ moieties is considered to improve the Li^+^ ion transfer between the active material and polymer electrolyte that lead to a better performance at high C-rates^47,48^. It is worth noting that it is quite hard to achieve good quality of electrode using unmodified CNT. In fact, large agglomerates of carbon are obtained, as shown with the SEM image of Supplementary Fig. S1, even with intensive mixing in solvents or through mechanical mixing. Thus, the corresponding electrode was not fabricated. Additional electrochemical results for electrodes made with unmodified and grafted-VGCF carbons (see Supplementary Fig. S5) and with modified Ketjen carbon powders (see Supplementary Fig. S6) are presented in the Supporting Information.

Hence, carbon engineering is key to optimize the cathode formulation and boost the electrochemical performance of composite electrodes^45^.

## Discussion

We have demonstrated that using self-generated Li-rich carbons improves the electrochemical performance of solid-state batteries for uniform material dispersion, better electrode quality, neutral NMC particle surface by eliminating basic impurities (LiOH, Li_2_CO_3_), filling micro- and meso-pores of carbon by organic molecules, and improved Li^+^ ions transfer at the carbon/active material interface^49^. Future works will focus on the grafting of different hydrophilic molecules such as those represented in Fig. 1. In addition, the amount of lithiated groups generated in contact with NMC will be determined using solid-state nuclear magnetic resonance (NMR) and inductively coupled plasma (ICP) analytical techniques. Moreover, the same method will be utilized with Ni-rich NMC cathode materials (e.g. LiNi_0.8_Co_0.1_Mn_0.1_O_2_, NCM811), which are also well adapted to the technique described in this work.

## Methods

### Synthesis of NTCDA-(aryl-NH_2_)_2_

According to a procedure previously described by our group^37^, dinitro-aryl-1,4,5,8-naphtalenetetra-carboxylic diimide molecule (NTCDA–(aryl–NO_2_)_2_) was first synthesized via the *N*-acylation reaction between 1,4,5,8-naphtalenetetra-carboxylic dianhydride (NTCDA) and 4-nitroaniline. Appropriate amount of the reagent was dissolved in 150 mL of anhydrous DMF followed by heating at 160 °C for 3 days until a clear brown color was obtained. The mixture was then filtered and washed with DMF and acetone by centrifugation until the supernatant was clear. The powder was dried overnight in an oven at 100 °C and then dispersed in 100 mL ethanol. A distillation column was placed on top and the mixture was purged in a nitrogen flow. The temperature was set to 110 °C. FeCl_3_·6H_2_O and iron powder were dispersed in a mixture of ethanol and glacial acetic acid, and then added dropwise to the flask over several minutes. After 2 days of reaction, the remaining Fe^0^ powder was recovered using strong magnets and the solution was filtered. The resultant solid was thoroughly washed with deionized water until an approximate pH of 7 was obtained. After a final wash with acetone, an orange powder was obtained with a 95% yield.

### Synthesis of modified carbons

#### Electrochemical exfoliation of graphite: EG

Graphene powder was obtained using electrochemical exfoliation of a graphite foil, as reported by Delaporte et al*.*, but with certain protocol modifications^18^. An anode of graphite (a thin foil) and a Pt mesh cathode were immerged in 0.1 M H_2_SO_4_ and connected to a DC power supply. The electrochemical exfoliation was performed by applying a DC voltage of 3 V between the two electrodes. After approximately 3 h of electrolysis, the electrodes were carefully removed and the dark solution was filtered. The resulting powder was washed several times with Nanopure water to remove residual acid. After drying at 80 °C for several days, a dark grey powder (EG) was obtained.

#### Synthesis of water-soluble carbons: VGCF-COOH and CNT-COOH

The modification procedure was adapted from a recent work^38^. 5 g of carbon (VGCF or CNT, see Fig. 1) was dispersed in a 100 mL 0.5 M H_2_SO_4_ aqueous solution using an ultrasonic tip for approximately 30 min. Concurrently, a 0.01 equivalent *p*-substituted aromatic amine with –COOH groups was dissolved in a 100 mL of deionized water until transparent. The two mixtures were then combined and bulled with nitrogen gas for 10 min. A 0.03 equivalent of sodium nitrite compared with carbon was gradually added to the mixture in a continuous nitrogen flow to generate the corresponding aryl diazonium ions (N_2_^+^–aryl–COOH). The temperature was set to 95 °C and the mixture was allowed to react overnight. After the reaction was complete, the mixture was vacuum-filtered using a Büchner assembly and nylon filter with a 0.22 µm pore size. Then, the black paste was thoroughly dispersed in deionized water for 2 h and centrifuged to remove the upper part of the liquid. This step was repeated until the liquid attained an approximate pH value of 7. Finally, the powder was successively washed with DMF and acetone, and vacuum-dried at 120 °C for approximately two days before being utilized. The modified VGCF and CNT carbons are VGCF–COOH and CNT–COOH, respectively.

#### Synthesis of redox-active carbons: grafted-Ketjen-orga and grafted-Ketjen-acid

For the synthesis of modified Ketjen Black carbon with NTCDA–(aryl)_2_ groups, acidic and organic media were used. Depending on the solvent used and presence or absence of acid, the kinetics of the reaction and yield of grafted groups are greatly impacted^50^. As explained before, for the synthesis of water-soluble carbons, 1 g of Ketjen Black carbon was dispersed and sonicated. Then, a quantity of NTCDA–(aryl–NH_2_)_2_ molecules, corresponding to a 0.1 equivalent compared with the carbon, was added in the flask and stirred vigorously in nitrogen flow. To facilitate the reaction, 10 equivalents of diazotization reagent (NaNO_2_ for acidic medium and Tert-butyl nitrite for acetonitrile solution) in comparison with the amine were gradually added to the mixture. The reaction time was two days at an ambient temperature and at 95 °C, when acetonitrile and 0.5 M HCl were utilized, respectively. After the reaction was complete, the powders were washed by filtration and centrifugation except for the materials prepared in acetonitrile, for which washing with deionized water is not necessary. The powders are then vacuum-dried at 120 °C for approximately two days before being utilized. The Ketjen Black carbons modified in organic and acidic media are grafted-Ketjen-orga and grafted-Ketjen-acid, respectively.

### Preparation of NMC@carbon composites

For the preparation of NMC@carbon composites, certain amount of carbon was considered corresponding to 0.5 wt% of LiNi_0.6_Mn_0.2_Co_0.2_O_2_ (NMC622, Hydro-Quebec) when EG and CNT–COOH were used, and 1 wt% when VGCF–COOH was utilized. The carbon was dispersed in deionized water using an ultrasonic tip until stable suspension was obtained, as shown in Supplementary Fig. S1 for CNT–COOH. Then, NMC powder was added to the solution followed by heating at 80 °C with vigorous stirring for 30 min. The water was quickly removed using a rotary evaporator and resulting black powder was directly vacuum dried at 120 °C for 2 days. When EG, VGCF–COOH, and CNT–COOH are used in the preparation of the cathode composite, the resulting materials obtained were NMC@EG, NMC@VGCF–COOH, and NMC@CNT–COOH, respectively.

### Characterization

Thermogravimetric analysis was conducted using a TGA 550 model (TA instruments) with a heating rate of 5 °C min^−1^ and air flow of 90 mL min^−1^ from 30 to 700 °C.

FTIR spectrum of NTCDA–(aryl–NH_2_)_2_ molecule was obtained using a Bruker Vertex 70 spectrometer equipped with a smart ATR accessory.

EG powder was characterized using X-ray diffraction (XRD) by a Philips X'Pert diffractometer θ–2θ with Cu Kα1,α2 radiation (λ1 = 1.5405 Å, λ2 = 1.5443 Å) and a monochromator to avoid Kβ radiation. The data were collected between 5° and 60° using a 0.02° step and an integration time of 1.3 s per step with an X'Celerator detector.

Micro-Raman surface analyses of pristine NMC and NMC@CNT–COOH powders were performed using a HORIBA LabSpec 5 apparatus with a 532 nm laser excitation wavelength.

Adsorption isotherms were measured using a QuadraSorb Station 3 instrument (version 5.04, Quantachrome Instrument). The porous texture of the pristine Ketjen and grafted-Ketjen-acid carbons was characterized using nitrogen as adsorbent at 77.3 K. The volume of gas adsorbed was recorded for relative pressures (P/P_0_) ranging from 4 × 10^–2^ to 1. The N_2_ adsorption data were used to calculate the BET specific surface area and total pore volume. The pore size distribution was calculated through simulation of the isotherm using density functional theory (DFT) and Monte-Carlo calculations.

The chemical composition of the pristine Ketjen and grafted-Ketjen-acid carbon surfaces (5 nm deep) was investigated by X-ray Photolectron Spectroscopy (XPS) using a PHI 5,600-ci spectrometer (Physical Electronics, Eden Prairie, MN). The main XPS chamber was maintained at a base pressure of < 8.10^–9^ Torr. A standard aluminum X-ray source (Al kα = 1,486.6 eV) was used to record the survey spectra (1,400–0 eV, 10 min), while magnesium was used to obtain high-resolution spectra—both without charge neutralization. The detection angle was set at 45° with respect to the normal of the surface and the analyzed area was 0.5 mm^2^. High-resolution spectra were obtained for C 1s and O 1s with 30 and 20 sweeps, respectively.

NTCDA–(aryl–NH_2_)_2_ molecule, EG and NMC@VGCF–COOH powders were observed with a FlexSEM 1000 Scanning Electron Microscope (Hitachi High-Technologies Corporation) placed in a dry room. Secondary electron (SE) images were obtained at an accelerating voltage of 5 kV and a working distance of approximately 5–6 mm. Tescan Mira SEM was used to evaluate structural morphology of NMC@CNT, NMC@CNT–COOH and NMC@EG cathode composites.

Confocal images of NMC electrodes were acquired with a 3D confocal microscope (VK-X200, Keyence Laboratories) to measure the roughness of the cathode surface. Two objective lens magnifications of × 20 and × 50 were used. An image mosaic of 3 × 3 images were acquired. The roughness was evaluated by the arithmetic mean roughness RA (average of the absolute value along the reference length) from 3D mosaic by a series of images at different focus. The mosaic width at × 20 and × 50 magnifications is 2027.52 µm and 798.72 µm, respectively.

TEM images of the NMC@CNT–COOH cathode composite were obtained using a HF3300 microscope (Hitachi High-Technologies Corporation) operating at 300 kV. Chemical composition was assessed with an EELS GIF Quantum system from Gatan Company. Samples were dispersed in ethanol and drop casted on a QUANTIFOIL Holey Carbon Films prior to the analysis.

### Preparation of electrodes

#### Modified and unmodified carbon electrodes

For the preparation of carbon electrodes, 50 wt% of unmodified Ketjen or grafted-Ketjen-acid powders were mixed with 50 wt% of PVDF binder in n-methyl-2-pyrrolidone (NMP) solvent. The solution was mixed with a Thinky mixer until a homogenous solution was obtained with an appropriate viscosity for coating. The obtained slurry was then spread on an aluminum current collector (15 μm) and allowed to dry for a few minutes under a ventilated fume hood. The electrode was further dried at 50 °C for 1 h and at 75 °C for approximately 12 h. The carbon electrode was punched with a diameter of 16 mm and dried under vacuum at 120 °C for 24 h before it was assembled in a coin-cell (CR2032).

#### NMC polymer electrodes

NMC powder (pristine powder or modified NMC@carbon composite) and carbon black (pristine powder or modified carbon) were mixed with the PEO-based polymer electrolyte (Hydro-Quebec) solution in a mixed solvent of acetonitrile and toluene (80:20 v/v). A total amount of 2 wt% of carbon was used for all the electrodes and when two pristine carbons are used in the composition of the electrode (e.g. Ketjen + VGCF), 1 wt% of each was employed. The slurry was cast on a carbon-coated aluminum foil (15 µm) to have a loading of 8 mg/cm^2^ after drying. The process was conducted inside a dry room with a dew point less than − 50 °C. The electrodes were conserved in a metal plastic bag in dry atmosphere before being utilized.

### Cells assembly

#### Carbon electrodes in Li-ion half-cells

First, the electrodes are dried overnight at 120 °C in vacuum. The coin cells were assembled using the electrode synthesized using pristine Ketjen and grafted-Ketjen-acid, Li metal foil (40 µm, Hydro-Quebec) as a counter and reference electrode, a separator (Celgard-3501), and 1 M LiPF_6_ in ethylene carbonate (EC):diethyl carbonate (DEC) (3:7 v/v) electrolyte in an argon-filled glove-box (O_2_ < 5 ppm).

#### All-solid-state Li metal polymer batteries

First, the NMC cathode electrodes were dried at 80 °C in vacuum for approximately 12 h. The PEO-based polymer electrolyte film (Hydro-Quebec) with 25 µm thickness was employed as a solid polymer electrolyte (SPE) without using an additional separator. The SPE film was placed between the cathode electrode and Li metal foil (35 µm, Hydro-Quebec) and laminated at 80 °C to assemble the coin-type cells (CR2032). The diameter of cathode electrode was 16 mm. The process was performed inside a dry room having a dew point less than − 50 °C or inside the Ar-filled glove box (H_2_O, O_2_ < 5 ppm).

### Electrochemical testing

#### Cyclic voltammetry

Cyclic voltammetry (CV) was conducted using VMP3 potentiostat (BioLogic Science Instruments) for unmodified Ketjen and grafted-Ketjen-acid cathodes in comparison between 1.8–2.8 V vs. Li/Li^+^ at a scan rate of 0.03 mV s^−1^.

#### Galvanostatic cycling

Galvanostatic cycling experiments of Li/SPE/NMC batteries were conducted at an operating temperature of 50 °C (± 0.5 °C) at different C rates (nC rate: reaction of one Li^+^ in n hours) with the potential window ranging from 2.75 to 4.2 V (vs. Li/Li^+^). The cell was kept at 50 °C for ∼4 h prior to the cycling to ensure temperature uniformity.

## Supplementary information

Supplementary Information
