# Supplementary material for: Facile formulation and fabrication of the cathode using a self-lithiated carbon for all-solid-state batteries
Source: Sci Rep. 2020 Jul 16;10:11813. doi: 10.1038/s41598-020-68865-8 (PMC7367347; doi:10.1038/s41598-020-68865-8)
Supplement: Supplementary file 1 — Supplementary Information [file 41598_2020_68865_MOESM1_ESM.docx]

**Supplementary materials**

**Facile Formulation and Fabrication of the Cathode Using a**

**Self-Lithiated Carbon for All-Solid-State Batteries**

**N. Delaporte**^‡§^**, A. Darwiche**^‡^**, M. Léonard**^‡^**, G. Lajoie**^‡^**, H. Demers**^‡^**, D. Clément**^‡^**, R. Veillette**^‡^**, L. Rodrigue**^‡^**, M. L. Trudeau**^‡^**, C. Kim**^‡^**, K. Zaghib**^‡*^

‡ Hydro-Québec, Center of Excellence in Transportation Electrification and Energy Storage, Varennes, Québec, J3X 1S1, Canada.

Corresponding author: *[zaghib.karim@hydro.qc.ca](mailto:zaghib.karim@hydro.qc.ca); §delaporte.nicolas@hydroquebec.com

**S1. SEM observations of NMC/carbon composites**


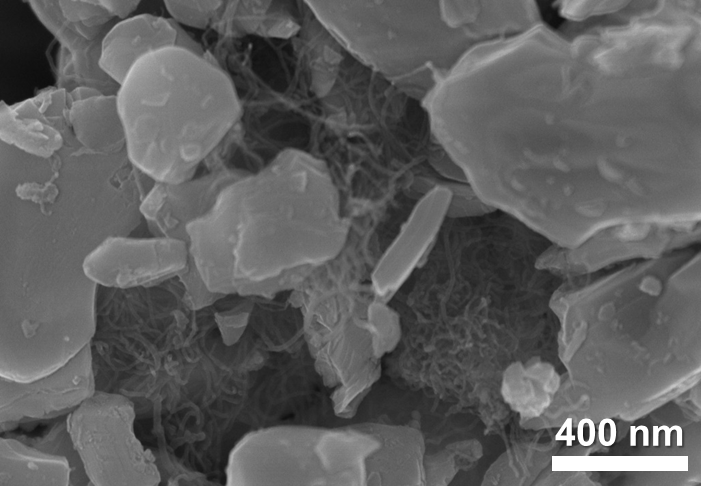


**Fig. S1.** SEM image of NMC/CNT electrode composite made by mixing the pristine NMC powder with unmodified CNT.


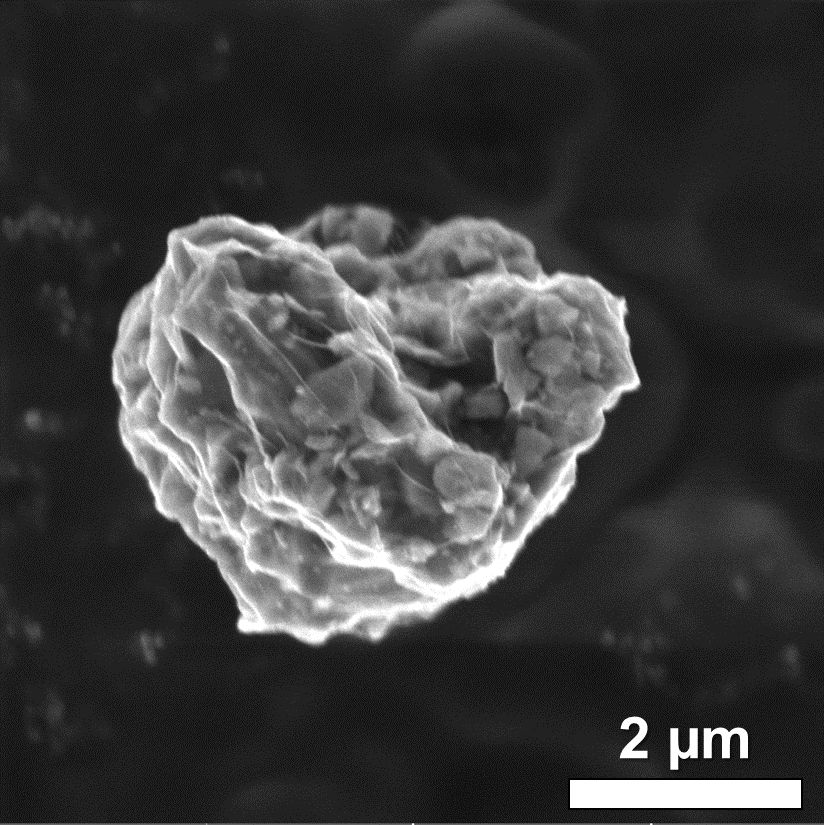


**Fig. S2.** SEM image of NMC@EG powder. The graphene sheet covered the entire surface of the NMC particles.

**S2. Characterization of NTCDA–(aryl–NH_2_)_2_ and grafting of NTCDA–(aryl)_2_ moieties on Ketjen carbon**

Additional cathode formulations were realized by employing a Ketjen Black carbon modified with NTCDA–(aryl)_2_ groups. We recently reported the potential of such carbons for energy storage applications owing to the ability of these organic groups to reversibly store lithium ions ^1^. Fig. S1 shows the schematic representation of the grafting reaction of NTCDA–(aryl)_2_ moieties on Ketjen Black carbon. The first step is to generate the corresponding highly unstable NTCDA–(aryl-N_2_^+^)_2_ diazonium ions in a solution in the presence of carbon. The reaction was performed either in acidic or organic media. Following the two possible mechanisms ^2^, the reduction of diazonium ions led to the grafting of the redox groups on the surface of Ketjen Black via strong C–C bonds. This carbon can be used as a pseudo-capacitor material to rapidly store energy in a few seconds or minutes owing to its highly conjugated structure facilitating electron de-localization and redox reactions at approximately 2.45 V vs. Li/Li^+^.

**Fig. S1.** Schematic representation of the grafting reaction of NTCDA–(aryl)_2_ moieties on Ketjen Black carbon through decomposition of *in-situ* generated NTCDA–(aryl–N_2_^+^)_2_ ions. The redox reaction taking place at 2.45 V vs. Li/Li^+^ is also represented with the uptake/release of two electrons per grafted molecule.

Fig. S2a shows a SEM image of the as-synthesized NTCDA–aryl–(NH_2_)_2_ powder that has small orange rods with an oval-like structure and a uniform size of ~5 μm. After grafting, no such rods are observed in the Ketjen Black carbon powder confirming the efficacy of surface grafting. In addition, the grafting of these molecule considerably reduced the agglomeration of carbon, which is key for achieving a smooth electrode with well dispersed carbon and active material ^1^. The redox molecule was also characterized by FTIR analysis and its corresponding spectrum is shown in Fig. S2b. The structure of the molecule was confirmed by the typical absorption bands attributed to the stretching vibrations of C=O (1710, 1620, and 760 cm^-1^), C–H bonds of the aromatic rings (3080 and 985 cm^-1^), C–N stretching vibrations of imide function (1660 and 1350 cm^-1^), stretching vibration of –NH_2_ function (3300–3400 cm^-1^), and absorption band at approximately 1580 cm^-1^ that is associated to the stretching vibration of naphthalene ring skeleton.

**
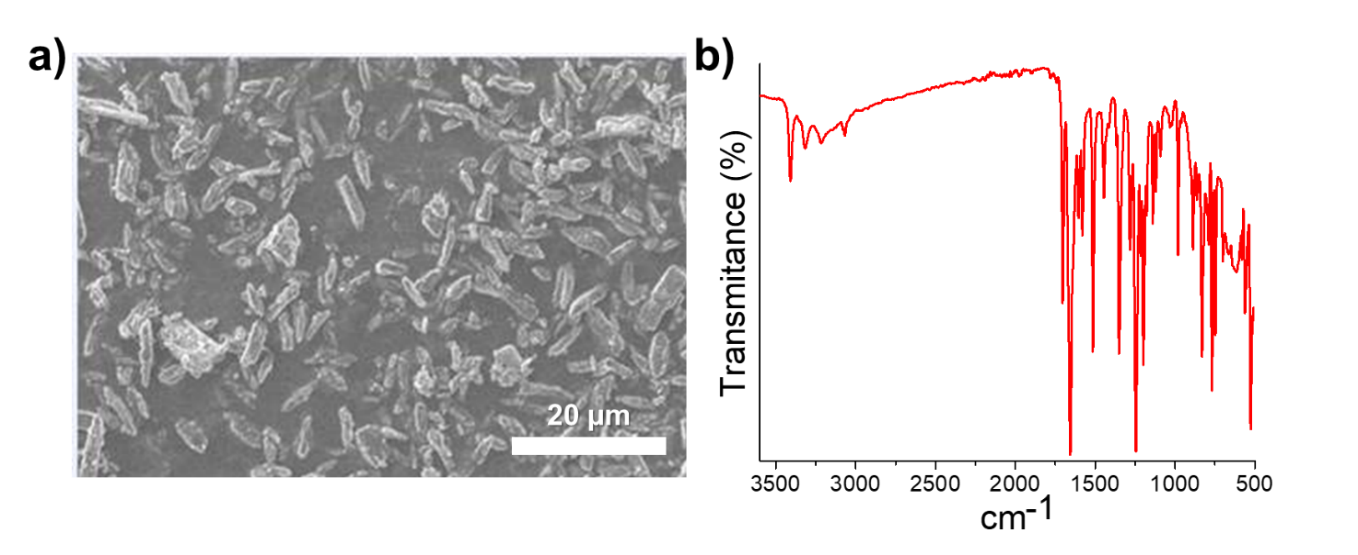
**

**Fig. S2.** a) SEM image and b) FTIR spectrum of NTCDA–(aryl–NH_2_)_2_ molecule.

The presence of NTCDA–(aryl)_2_ groups on the surface of Ketjen Black carbon was verified by XPS analyses. Fig. S3 presents the XPS a), b) C 1s and c), d) O 1s core level spectra for a), c) grafted-Ketjen-acid and b), d) pristine Ketjen Black carbon powders. Both carbons showed a peak at ~285.0 eV attributed to the different carbon bonds C–C, C–H or C=C. In addition, small contributions at 286 and 287 eV were observed and associated to C–O/C–N and C=O bonds, respectively. The two signals were more intense for grafted-Ketjen-acid sample due to the presence of a nm-thick layer of NTCDA–(aryl)_2_ groups on the surface. This was confirmed by the O 1s core level spectrum of the grafted carbon (see Fig. S3c) showing a large peak centered at ~532 eV corresponding to C=O bonds of grafted organic moieties, while the spectrum for the unmodified Ketjen Black carbon showed background noise.

**
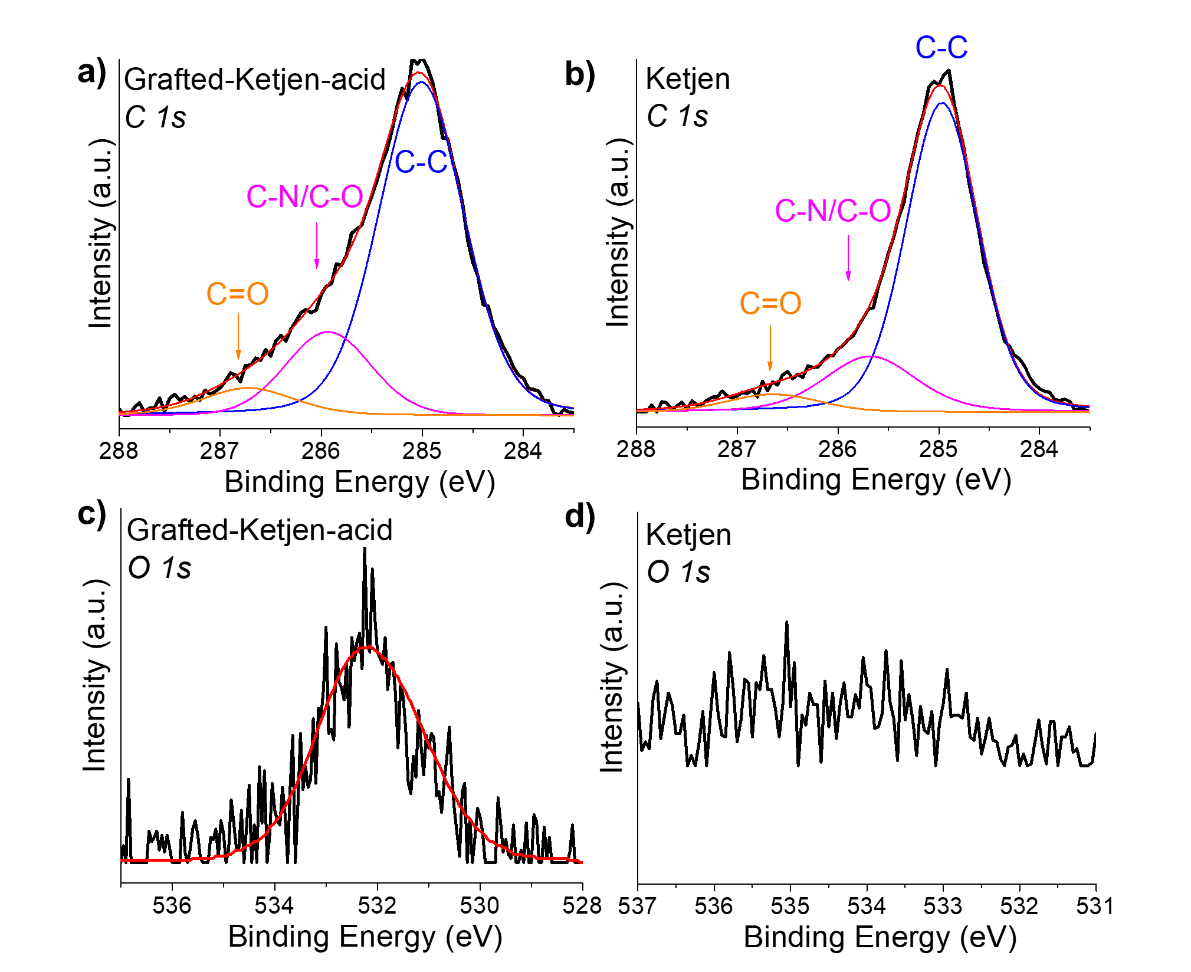
**

**Fig. S3.** a), b) XPS C 1s and c), d) O 1s core level spectra for a), c) grafted-Ketjen-acid and c), f) unmodified Ketjen Black carbon powders.

Nitrogen adsorption-desorption isotherms obtained at 77 K for the grafted-Ketjen-acid (▲) and unmodified carbon (■) powders are presented in Fig. S4a. The shape of isotherms is conventional of an extended microporous structure (type I isotherm), and it was found that this carbon is dominated by two types of pores: smaller than 1 nm (microporosity) and those comprised between 1.5–3.5 nm (small mesopores). After modification, a drop of the BET specific surface area from 1238 to 600 m^2^.g^-1^ was calculated, which is found to be consistent with the obstruction of small pores by the grafted NTCDA–(aryl)_2_ moieties. The microporous volume was divided twice for grafted-Ketjen-acid carbon (0.29 cm^3^.g^-1^) in comparison with the unmodified Ketjen Black (0.6 cm^3^.g^-1^). This is because the smaller quantity of micro and mesopores limits the quantity of gas and solvents adsorbed, which are known to create additional porosity in the cathode during drying and definitively lead to a loss of electrical contact in the electrode. Concurrently, the outer surface of Ketjen Black modified with the NTCDA–(aryl)_2_ groups induces a better dispersion of carbon in the cathode owing to better affinity with the polymer (oxygen-rich surface, see XPS results of Fig. S3). Here, contrary to the grafting of aryl–COOH groups (see Fig. 1 in the manuscript for mechanism), the NTCDA–(aryl)_2_ groups in contact with NMC in water are not spontaneously lithiated and a premix is not necessary. However, they are electrochemically active at 2.45 V vs. Li/Li^+^. The cyclic voltammograms for the grafted-Ketjen-acid (---) and unmodified carbon (—) electrodes are shown in Fig. S4b. A pure capacitive behavior was obtained for pristine carbon showing a conventional I-V rectangular shape. In addition, a reversible redox process at approximately 2.45 V vs. Li/Li^+^ associated with the release/uptake of two Li^+^ ion was observed (see Fig. S1). Considering the faradic contribution of the redox peak (~34.7 mAh.g^-1^) and theoretical capacity for NTCDA–(aryl)_2_ moieties of ~128.9 mAh.g^-1^, a grafting loading of 26.9 wt. % can be estimated, which corresponds with the grafted amount of aryl–COOH groups on CNTs (~33%, see Fig. 2 in the manuscript). Although the redox potential of NTCDA–(aryl)_2_ groups is too low to permit their lithiation in the potential window where the NMC cathode is cycled (2.75–4.2 V), we demonstrated that using this carbon produces a better electrode quality and reduces the porosity during the drying step. Consequence, superior electrochemical performance is expected in comparison with the electrode using commercial Ketjen Black (Fig. S6).

**
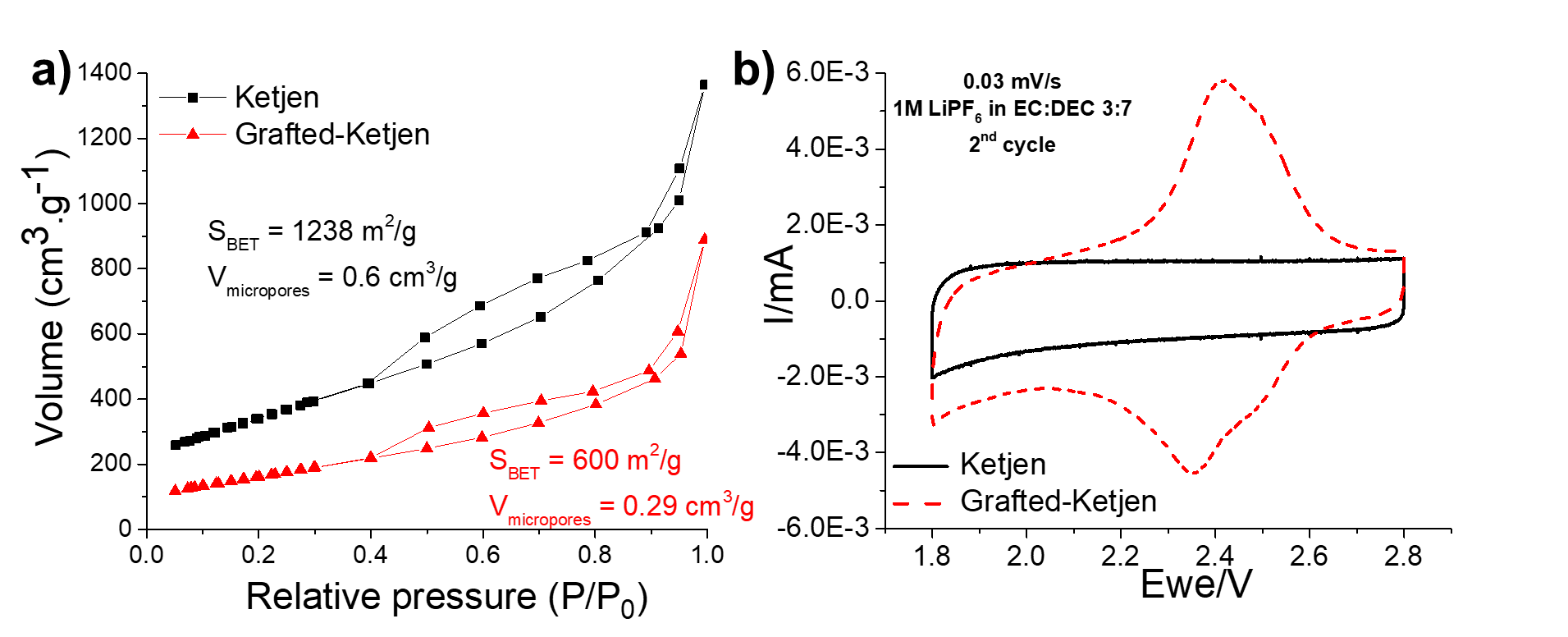
**

**Fig. S4.** a) Nitrogen adsorption-desorption isotherms at 77 K for grafted-Ketjen-acid (▲) and unmodified carbon (■) powders. b) Cyclic voltammograms (2^nd^ cycle) for grafted-Ketjen-acid (---) and unmodified carbon (—) electrodes recorded in 1 M LiPF_6_ EC:DEC 3:7 electrolyte at a scan rate of 0.03 mV.s^-1^.

**S3. Electrochemical performance of various composite electrodes**

Fig. S5a shows the conventional charge/discharge profiles at a cycling rate of C/24 for three NMC electrodes synthesized with: NMC and Ketjen Black (—), NMC and a mix of Ketjen Black and VGCF carbons (---), and NMC@VGCF-COOH composite with Ketjen Black (‧‧‧‧). As discussed in the manuscript (Fig. 9), the higher discharge capacity for the blank electrode (the anticipated capacity for NMC622 is approximately 160 mAh.g^-1^ at 4.2 V cut-off vs. Li/Li^+^) could be by an underestimation of the active material mass owing to the problems of homogeneity in the electrode and presence of agglomerates. Fig. S5b shows the corresponding rate capability for the same electrodes. First, 50 wt. % of the mass of Ketjen Black was substituted with a pristine VGCF carbon for the electrode labelled NMC + Ketjen Black + VGCF (●). Better discharge capacities are obtained when the cycling rate is increased in comparison with the blank electrode (■), which is expected due to the good electronic conductivity of this kind of graphitized carbon. When the NMC@VGCF–COOH composite (▲) is used, superior performance are obtained for C-rates ranging from C/12 to C/2 because of the optimized dispersion of carbon and active material in the cathode (see Fig. 5 and 6 in the manuscript).

**Fig. S5.** a) Galvanostatic charge/discharge profiles at a cycling rate of C/24 and b) rate capability of three NMC electrodes synthesized with: NMC and Ketjen Black (—,■), NMC and a mix of Ketjen Black and VGCF carbons (---,●), and NMC@VGCF-COOH composite with Ketjen Black (‧‧‧‧,▲).

Additional electrochemical tests were realized with different Ketjen Black carbons modified with NTCDA–(aryl)_2_ groups (Fig. S6). The cathode labelled NMC + grafted-Ketjen-org (---,●) shows a clear enhancement of the electrochemical performance in comparison with the bare electrode using unmodified Ketjen Black as a conductive additive (—,■). However, as discussed, the discharge capacity at C/24 is higher than the anticipated capacity of NMC622 material indicating an underestimation of the active material. Consequently, the real capacities for the NMC + grafted-Ketjen-org electrode are probably slightly lower than the value reported in Fig. S6b (●). In conclusion, the use of modified-Ketjen Black carbons helps to increase the dispersion of cathode materials in the electrode, but it appears to be less efficient than the NMC@carbon composites. The electrodes synthesized with the NMC@VGCF–COOH composite and grafted-Ketjen-org (▲) or grafted-Ketjen-acid (**▼**) carbons, delivered a specific capacity of approximately 150 mAh.g^-1^ at C/24, and demonstrated better discharge capacities at high C-rates. The best performance was observed with the grafted-Ketjen-acid carbon because of its high grafted NTCDA–(aryl)_2_ moieties in comparison with the carbon synthesized in organic medium (grafted-Ketjen-org) ^1^.

**Fig. S6.** a) Galvanostatic charge/discharge profiles at a cycling rate of C/24 and b) rate capability of four NMC electrodes synthesized with: NMC and Ketjen Black (—,■), NMC and grafted-Ketjen-org (---,●), NMC@VGCF-COOH composite and grafted-Ketjen-org (‧‧‧‧,▲), and NMC@VGCF-COOH composite and grafted-Ketjen-acid (---,**▼**).

**References**

1. Delaporte, N., Belanger, R. L., Lajoie, G., Trudeau, M. & Zaghib, K. Multi-carbonyl molecules immobilized on high surface area carbon by diazonium chemistry for energy storage applications. *Electrochimica Acta* **308**, 99–114 (2019).

2. Delaporte, N., Zaghib, K. & Bélanger, D. In situ formation of bromobenzene diazonium ions and their spontaneous reaction with carbon-coated LiFePO_4_ in organic media. *New J. Chem.* **40**, 6135–6140 (2016).
